# Clinical application of indocyanine green fluorescence navigation technique in laparoscopic common bile duct exploration for complex hepatolithiasis

**DOI:** 10.1186/s12893-024-02411-5

**Published:** 2024-04-20

**Authors:** Wenfei Wang, Sanli Feng, Zhuang Li, Zhenyu Qiao, Liusheng Yang, Lin Han, Fei Xu, Xiangyu Dong, Minghui Sheng, Dengqun Sun, Yanjun Sun

**Affiliations:** 1 Department of general surgery, The Chinese People’s Armed Police Forces Anhui Provincial Corps Hospital, Hefei, 230041 China; 2 2The First Affiliated Hospital of Anhui University of Chinese Medicine, Hefei, China

**Keywords:** Indocyanine green, Laparoscopy, Bile duct injury, Common bile duct exploration

## Abstract

**Background:**

This study investigated the clinical application of the indocyanine green (ICG) fluorescence navigation technique in bile duct identification during laparoscopic common bile duct exploration (LCBDE) for complex hepatolithiasis.

**Methods:**

Eighty patients with complex hepatolithiasis were admitted to our department between January 2022 and June 2023 and randomly divided into control and observation groups. The control group underwent conventional LCBDE, while the observation group underwent LCBDE guided by ICG fluorescence.

**Results:**

Intraoperatively, the observation group had shorter operation and search times for the common bile duct (CBD), as well as reduced intraoperative blood loss and fewer complications, such as conversion to laparotomy and various injuries (gastroduodenal, colon, pancreatic, and vascular) than the control group, with statistical significance (*P <* 0.05). Postoperatively, the observation group had lower rates of postoperative bile leakage, abdominal infection, postoperative hemorrhage, and residual stone than the control group. Additionally, the observation group demonstrated significantly shorter times for resuming flatus, removal of the abdominal drainage tube, and hospitalization than the control group, with statistical significance (*P <* 0.05).

**Conclusion:**

ICG fluorescence navigation technology effectively visualizes the bile duct, improves its identification rate, shortens the operation time, prevents biliary tract injury, and reduces the occurrence of complications.

## Introduction

Laparoscopic common bile duct exploration (LCBDE) is the primary surgical method to treat common bile duct (CBD) stones, as it is minimally invasive and promotes fast recovery [1]. However, it can be challenging to perform surgery in complex biliary patients with a history of multiple upper abdominal surgeries and severe cholangitis, with a high risk of bile duct injuries (BDI). Indocyanine green (ICG) is a laparoscopic imaging agent that fluoresces and is excreted exclusively in the bile by the liver after uptake, allowing identification of bile duct anatomy. However, the clinical utility of fluorescent biliary imaging during laparoscopic cholecystectomy (LC) has been recognized and proven to be safe and reliable [2, 3]. There is limited research on ICG in LCBDE, particularly evaluating its clinical efficacy in complex hepatolithiasis surgery. Therefore, this study retrospectively analyzed the clinical data of 80 patients who underwent LCBDE with ICG fluorescence navigation to confirm the feasibility and safety of ICG use in LCBDE and explore its potential application value.

## Materials and methods

### General Information

This study included 80 patients with complex hepatolithiasis who underwent LCBDE at our hospital between January 2022 and June 2023. The inclusion criteria were as follows: a history of at least one previous hepatobiliary or other upper abdominal surgery, pre-operative imaging diagnosis of intrahepatic and extrahepatic bile duct stones with an assessment of stone distribution, no history of ICG allergy, and the ability to tolerate laparoscopic surgery and general anesthesia. Conversely, patients were excluded if they had no previous history of CBD exploration or other abdominal surgeries, inability to tolerate laparoscopic surgery and general anesthesia due to poor physical condition, easy visualization of the CBD under laparoscopy, or allergy to ICG. Patients who agreed to participate in the study and met the inclusion criteria were randomly divided into the control group (*n* = 40) and the observation group (*n* = 40) at a ratio of 1:1. Randomization was conducted through a computer-generated random sampling number list (https://randomizer.org). This study was approved by the Ethics Committee of the Chinese People’s Armed Police Forces Anhui Provincial Corps Hospital. All patients and their families provided informed consent by signing a Consent Form.

### Methods

The surgeries in the control and observation groups were performed by the same medical team. The control group underwent conventional LCBDE, following standard procedures. Patients were positioned in a right-side-up supine position under general anesthesia with endotracheal intubation. A 10 mm incision was made below the umbilicus to insert a trocar as an observation port and establish pneumoperitoneum, followed by inserting a 30° laparoscope. The intraabdominal cavity was explored, and trocar ports were established based on the presence of intra-abdominal adhesions. The adhesions were lysed under direct vision, and appropriate ports were placed for surgery. The primary operating port was inserted below the xiphoid process on the right side of the falciform ligament, and an auxiliary operating port was placed along the right axillary line. Additional auxiliary ports were added as necessary. The CBD was identified, and a cholangioscope was used to explore and remove the stone, followed by a T-tube for drainage after the obstruction was relieved. A subhepatic drainage tube was also inserted. Three months later, sinus tract angiography and cholangioscopy were performed, and the T-tube was removed depending on the presence of residual stones.

**Fig. 1:**
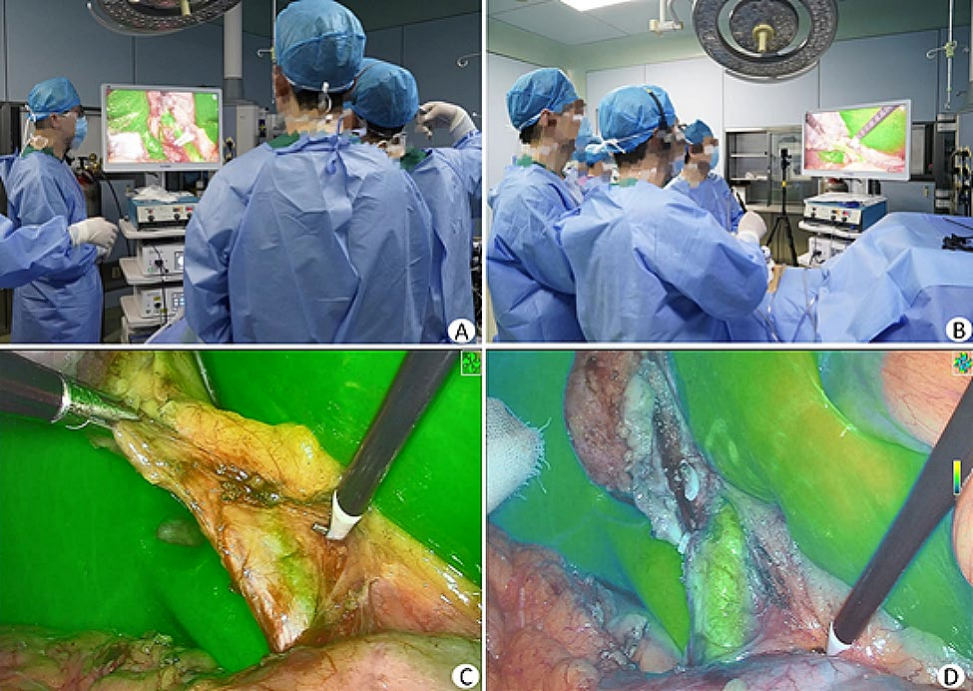
Intraoperative photographs of LCBDE under fluorescence navigation. **A-B** Intraoperative photograph; **C** Inflammation and edema of the gallbladder triangle made identification of the CBD difficult, but visualization of the CBD was achieved under fluorescence mode; **D** The CBD was separated under fluorescence navigation

**Fig. 2:**
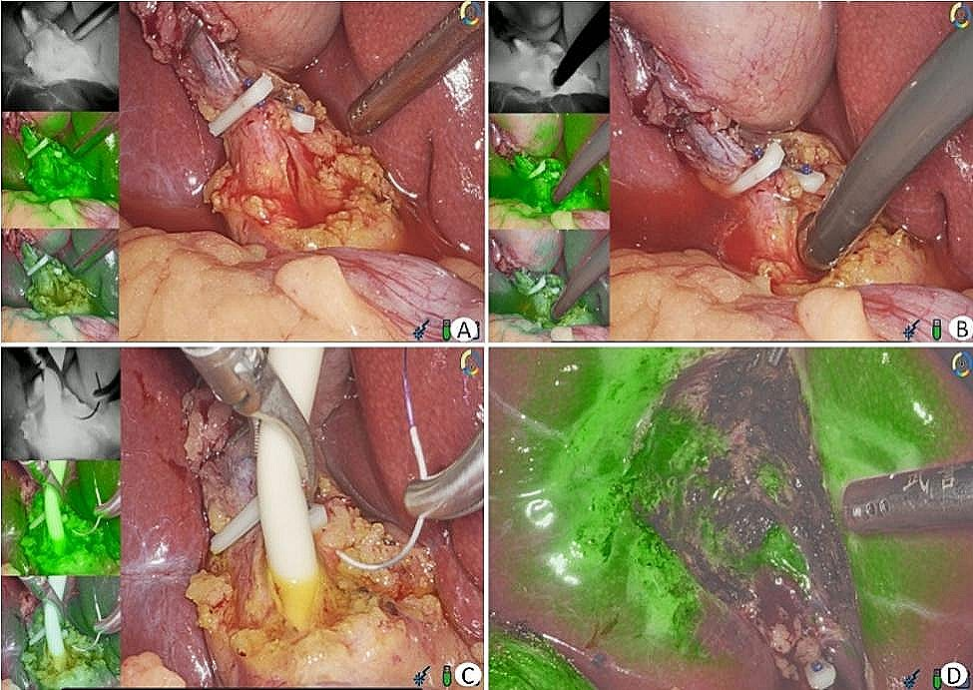
LCBDE under fluorescent navigation. **A** The CBD was incisioned; **B** Cholangioscopy was performed to explore and remove stones; **C** A T-tube was placed and sutured for fixation; **D** The gallbladder bed wound was examined to avoid bile leakage

According to Ishizawa et al. [3, 4], the commonly used approach and dose of ICG fluorescence cholangiography were adopted for the observation group. The standard concentration of ICG (1 mL, manufacturer: Dandong Yichuang Pharmaceutical Co., Ltd., approval number: National Medical Products Administration approval number H20055881, specification: 25 mg) was administered intravenously 30 min before surgery. The study employed the optoMedic fluorescence navigation endoscopy system (OPTO-CAM2100/LED2100, Guangdong, China). The subsequent surgical procedure was identical to that of the control group, with an additional step of identifying and separating the CBD under fluorescence guidance (Figs. 1 and 2).

### Observation indicators

Assessment of intraoperative and postoperative conditions is crucial to evaluate surgical outcomes. Intraoperative conditions such as operation time, time to locate the bile duct, and intraoperative blood loss were key factors. Additionally, the number of patients with conversion to open surgery, CBD, gastric and duodenal, colon, pancreatic, and vascular injuries should be meticulously documented. Conversely, postoperative conditions, including the number of patients with bleeding, abdominal infection, residual stones, bile leakage, gastrointestinal fistula, and pancreatitis, are critical indicators of surgical success. The time to removal of the abdominal drainage tube, resume flatus, and hospitalization time are also important postoperative measures. Notably, routine drainage tubes were placed in both groups postoperatively, and the drainage tube was removed when the drainage volume was less than 20 mL/day, with normal color. Postoperative bleeding or bile leakage was diagnosed based on observation of specific fluid characteristics in the drainage tubes.

### Statistical analysis

SPSS software (version 26.0) was used to statistically analyze the data. Measurement data were expressed as mean ± standard deviation (x̄±s) and compared between groups using independent sample t-tests. Count data were expressed as rates (%) and compared using the chi-square test. A P-value of less than 0.05 was considered statistically significant.

## Results

### Comparison of general data between the two groups

The general data for the observation group revealed a gender distribution of 16 males and 24 females, with an average age of 71.2 ± 10.4 years and a BMI of 22.5 ± 2.8 kg/m^2^. Regarding the history of hepatobiliary surgery, 23 cases had undergone surgery once, nine cases twice, and eight cases three times or more, with an average time elapsed since the last surgery of 20.5 ± 14.7 months. The CBD diameter was measured at 13.1 ± 3.3 mm, and the maximum diameter of stones was (10.1 ± 2.9) mm, with an average number of stones being 2.2 ± 1.3. Additionally, the total bilirubin, direct bilirubin, ALT, AST, ALP, and GGT levels were 59.8 ± 19.9 µmol/L, 38.7 ± 17.3 µmol/L, 60 ± 35.1 U/L, 71 ± 44 U/L, 127.9 ± 55.2 U/L, and 96.7 ± 42.1 U/L, respectively. The control group consisted of 19 males and 21 females, with an average age of 69 ± 10.7 years and an average BMI of 22.6 ± 3.4 kg/m^2^. Regarding hepatobiliary surgery history, 22 cases had undergone surgery once, 13 cases twice, and five cases three times or more, with a mean time elapsed since the last surgery of 18.1 ± 9.5 months. The CBD diameter was 12.4 ± 2.4 mm, and the maximum diameter of stones was 10.5 ± 2.8 mm, with an average number of stones being 2.3 ± 1.2. Moreover, the total bilirubin, direct bilirubin, ALT, AST, ALP, and GGT levels were 62.9 ± 22.8 µmol/L, 39.6 ± 19.4 µmol/L, 63.8 ± 30.3 U/L, 64 ± 34.5 U/L, 116.8 ± 50.2 U/L, and 82.1 ± 52.5 U/L, respectively. Furthermore, comparing general data between the two groups indicated no statistically significant differences (*P >* 0.05), demonstrating the comparability between the observation and control groups. Table 1 summarizes the findings.

**Table 1:** Comparison of general information between the two patient groups

Variables	Control group	Observation group	*P* values
Number of Cases	40	40	
Gender			0.652
male	19(47.5)	16(40)	
Female	21(52.5)	24(60)	
Age (years)	69.0 ± 10.7	71.2 ± 10.4	0.349
BMI	22.6 ± 3.4	22.5 ± 2.8	0.908
Number of Previous Biliary Surgeries			0.486
1	22(55)	23(57.5)	
2	13(32.5)	9(22.5)	
≥ 3	5(12.5)	8(20)	
Time since Last Surgery (months)	18.1 ± 9.5	20.5 ± 14.7	0.393
CBD Diameter (mm)	12.4 ± 2.4	13.1 ± 3.3	0.266
Total Bilirubin (umol/L)	62.9 ± 22.8	59.8 ± 19.9	0.508
Direct Bilirubin (umol/L)	39.6 ± 19.4	38.7 ± 17.3	0.827
ALT(U/L)	63.8 ± 30.3	60.0 ± 35.1	0.606
AST(U/L)	64.0 ± 34.5	71.0 ± 44.0	0.431
ALP(U/L)	116.8 ± 50.2	127.9 ± 55.2	0.353
GGT(U/L)	82.1 ± 52.5	96.7 ± 42.1	0.173
Maximum Stone Diameter (mm)	10.5 ± 2.8	10.1 ± 2.9	0.587
Number of Stones	2.3 ± 1.2	2.2 ± 1.3	0.722

BMI = Body Mass Index. CBD = Common bile duct. ALT = Alanine Amino transferase. AST = Aspartate aminotransferase. ALP = Alkaline phosphatase. GGT = Gamma-glutamyl transferase

### Comparison of intraoperative conditions between the two groups

No cases of ICG allergy were reported in either group. Furthermore, the observation group exhibited a significantly shorter operation time and decreased duration to locate the CBD than the control group. Moreover, the observation group had a lower incidence of conversion to open surgery and reduced intraoperative bleeding when compared to the control group. These statistically significant differences are evident in Table 2 (*P <* 0.05).

**Table 2:** Comparison of intraoperative conditions between the two patient groups

Variables	Control group	Observation group	*P* values
Number of Cases	40	40	
Operation Time (min)	181.5 ± 38.2	145.1 ± 35.0	<0.001
Time to Locate CBD (min)	48.4 ± 12.4	37.0 ± 8.5	<0.001
Blood Loss (ml)	218.2 ± 51.4	141.9 ± 42.5	<0.001
Conversion to Open Surgery	12(30)	3(7.5)	<0.05

### Comparison of intraoperative CBD Injury and other complications between the two groups

The incidence of intraoperative CBD injury was significantly lower in the observation group than in the control group (*P <* 0.05). Furthermore, the observation group exhibited a significantly lower incidence of other complications (3.125%) than the control group (8.75%) (*P* = 0.033). Table 3 provides the detailed information.

**Table 3:** Comparison of intraoperative common bile duct injuries and other complications between the two groups [n (%)]

Variables	Control group	Observation group	*P* values
CBD Injury	11(27.5)	4(10)	<0.05
Gastroduodenal Injury	3(7.5)	1(2.5)	>0.05
Colon Injury	2(5)	1(2.5)	>0.05
Pancreatic Injury	2(5)	1(2.5)	>0.05
Vascular Injury	7(17.5)	2(5)	>0.05
Total	14(8.75)	5(3.125)	<0.05

### Postoperative conditions of the two groups

Comparison between the control and observation groups revealed statistically significant differences in the incidence of postoperative bile leakage, abdominal infection, residual stones in the CBD, bleeding, time to resume flatus, and hospitalization duration (all *P <* 0.05, Table 4). However, no statistically significant variance was observed in the occurrence of postoperative pancreatitis and gastrointestinal fistula between the two groups (*P >* 0.05). Patients in both groups with complications, including bile leakage, abdominal infection, residual stones, bleeding, pancreatitis, and gastrointestinal fistula, received conservative treatment involving anti-infective therapy, nutritional support, and drainage, resulting in successful recovery. Notably, only one patient required a second surgery due to significant intra-abdominal bleeding post-initial operation, but the postoperative treatment was efficacious. Furthermore, the residual stones were removed using cholangioscopy, leading to successful patient recovery. All T-tubes were successfully removed after three months.

**Table 4:** Comparison of postoperative conditions between the two patient groups

Variables	Control group	Observation group	*P* values
Number of Cases	40	40	
Bile Leakage	11(27.5)	3(7.5)	<0.05
Abdominal Infection	9(22.5)	2(5)	<0.05
Residual Stones	12(30)	4(10)	<0.05
Gastrointestinal Fistula	4(10)	1(2.5)	0.356
Postoperative Bleeding	8(20)	1(2.5)	<0.05
Pancreatitis	3(7.5)	1(2.5)	0.608
Time of removal of abdominal drainage tube	7.6 ± 3.7	5.5 ± 2.3	<0.01
Time to resume flatus	2.0 ± 0.7	1.5 ± 0.6	<0.01
Hospitalization time	9.5 ± 3.7	7.4 ± 2.2	<0.01

## Discussion

BDI is an unavoidable challenge in biliary surgery, with the incidence of iatrogenic, attributed to the widespread application of laparoscopic hepatobiliary surgery. The incidence of LC-related iatrogenic BDI has been reported to be as high as 0.3–0.7% in foreign literature [5, 6]. Complex hepatolithiasis surgery typically involves re-exploration of the hepatobiliary tract due to prior hepatobiliary stone surgeries or other upper abdominal surgeries, such as gallbladder surgery (open cholecystectomy, recurrent stones after laparoscopic minimally invasive cholecystolithotomy, biliary stones after LC), open or laparoscopic CBD exploration with T-tube drainage, and other upper abdominal surgeries like gastric, duodenal, and transverse colon surgeries [7, 8]. Intra-abdominal adhesions, particularly severe adhesions of the hepatic portal tissues, are the primary challenge with repeat abdominal surgery because they can hinder the identification of the CBD during surgery, predisposing the patient to BDI and other injuries [9]. Hence, precise identification of bile ducts during surgery is imperative to prevent BDI. ICG fluorescence navigation technology has gained prominence in hepatobiliary surgery. ICG is a fluorescent dye that emits fluorescence when bound to proteins and exposed to near-infrared light, serving as a navigational aid during surgery. Following intravenous injection, ICG rapidly and completely binds to plasma proteins, albumin, and lipoproteins and is selectively absorbed by the liver and excreted via bile secretion [10, 11]. Its rapid excretion confers safety, minimal side effects, and non-interference in bodily reactions, thus making it advantageous [12]. ICG technology is becoming increasingly mature and is widely employed in the medical field. Its applications in hepatobiliary surgery encompass tumor localization, boundary delineation, liver staining segmentation, and visualization of biliary structures, thereby reducing surgical risks and complications [10, 13]. Notably, ICG fluorescent navigation technology has enabled real-time visualization of the biliary anatomy during LC and other complex biliary surgeries, thereby reducing the incidence of iatrogenic BDI [14, 15]. Furthermore, ICG facilitates clear visualization of biliary structures during procedures such as LCBDE, repeat CBD exploration, and complex hepatolithiasis surgeries, effectively reducing complications such as biliary duct injury and enhancing surgical efficiency due to its safety and high efficiency [3, 6, 10, 16].

In this study, we employed ICG fluorescent navigation technology to conduct LCBDE for complex hepatolithiasis. This advanced technology enables real-time visualization of the biliary system, eliminates the requirement for anatomical separation of the first hepatic portal and hepatoduodenal ligament, and facilitates efficient identification and separation of the bile ducts. Consequently, it improves bile duct recognition rates, reduces the need for conversion to open surgery, and enhances surgical precision by minimizing excessive dissection and reducing trauma to surrounding tissues, such as the gastrointestinal tract. Our results indicated that the observation group experienced significantly shorter times for locating bile ducts, operation duration, conversion to open surgery, bile leakage, abdominal infection, postoperative bleeding, and residual stone cases than the control group, with statistically significant differences (*P <* 0.05). Furthermore, the recovery time for bowel function, drainage tube removal, and hospital stay group were significantly shorter in the observation than in the control group, with statistically significant differences (*P <* 0.05). This indicates faster postoperative recovery and shorter hospital stay in the observation group. However, there were fewer gastrointestinal fistula and pancreatitis cases in the observation group than in the control group, without a statistically significant difference (*P >* 0.05), possibly due to the limited sample size. Nevertheless, this finding suggests that fluorescent laparoscopy can reduce gastrointestinal and pancreatic injuries to a certain extent. Over the past decade, ICG fluorescent navigation technology has advanced significantly in hepatobiliary surgery and has demonstrated its applicability in various other surgical procedures, such as lymph node dissection around gastrointestinal tumors, evaluation of colon anastomotic perfusion, bariatric surgery, urology, and gynecological surgeries [17]. However, ICG fluorescent imaging has certain limitations. First, the lack of unified standards and consensus on ICG dosage, concentration, administration route, and timing necessitates multi-center, large-sample studies. Second, a major drawback is the limited imaging depth of approximately 10 mm, resulting in poor visualization in cases where the thickness of adhesive tissue on the bile duct surface exceeds 10 mm [13]. The limitations of this study include being single-centered and having a relatively small sample size. We would recommend that a high-quality, multicenter, randomized clinical trial is developed specifcally to further evaluate the safety and effectiveness in this enriched population. Current research focuses on optimizing imaging technology to improve fluorescent navigation effects in complex hepatolithiasis surgeries [18].

## Conclusion

In summary, ICG navigation technology for LCBDE of complex hepatolithiasis is safe, feasible, convenient, and highly efficient. It enables intraoperative visualization of the CBD, mitigates the impact of adhesion and edema on structural recognition, reduces iatrogenic BDI and other complications, shortens operation times, and promotes postoperative recovery.

## Data Availability

The datasets used and analyzed during the current study available from the corresponding author on reasonable request.
